# Primary Healthcare managers’ understanding of institutional violence against dependent older adults

**DOI:** 10.1590/0034-7167-2024-0443

**Published:** 2025-10-03

**Authors:** Jonas Loiola Gonçalves, José Maria Ximenes Guimarães, Raimunda Magalhães da Silva, Girliani Silva de Sousa, Maria Cecília de Souza Minayo, Christina César Praça Brasil, Antônio Augusto Ferreira Carioca

**Affiliations:** I Universidade Estadual do Ceará. Fortaleza, Ceará, Brazil; II Universidade de Fortaleza. Fortaleza, Ceará, Brazil; III Universidade Federal de São Paulo. São Paulo, São Paulo, Brazil; IV Fundação Oswaldo Cruz. Rio de Janeiro, Rio de Janeiro, Brazil

**Keywords:** Elderly Person, Violence, Violence Against the Elderly, Primary Health Care, Health Managers, Persona Mayor, Violencia, Violencia Contra las Personas Mayores, Atención Primaria de Salud, Gestores de Salud

## Abstract

**Objectives:**

to analyze primary healthcare managers’ understanding regarding manifestations of institutional violence against dependent older adults in Brazil.

**Methods:**

a qualitative study, conducted in eight Brazilian cities. Interviews were conducted with 16 managers of primary healthcare services and specialized care units for older adults. To collect information, a semi-structured interview was used, with interpretation anchored in comprehensive and critical analysis.

**Results:**

institutional violence expresses the State’s lack of response through structural and organizational obstacles that enhance violence, and through healthcare professionals’ passivity or contempt towards dependent older adults, which is perpetuated by the lack or scarcity of provision of specific services.

**Conclusions:**

institutional violence against dependent older adults involves the State’s passivity, structural barriers, disarticulation of health production networks and lack of training or interest among professionals.

## RESUMEN

**Objetivos:**

analizar la comprensión de los gestores de Atención Primaria de Salud sobre las manifestaciones de violencia institucional contra las personas mayores dependientes en Brasil.

**Métodos:**

estudio cualitativo realizado en ocho ciudades brasileñas. Se entrevistaron a 16 gestores de servicios de Atención Primaria de Salud y unidades de atención especializada para personas mayores. Para la recopilación de información, se utilizó una entrevista semiestructurada, con una interpretación basada en un análisis exhaustivo y crítico.

**Resultados:**

la violencia institucional expresa la falta de respuesta del Estado a través de obstáculos estructurales y organizacionales que potencian la violencia, y a través de la pasividad o desprecio de los profesionales de la salud hacia las personas mayores dependientes, lo que se perpetúa por la falta o escasez de prestación de servicios específicos.

**Conclusiones:**

la violencia institucional contra las personas mayores dependientes implica la pasividad del Estado, barreras estructurales, desarticulación de las redes de producción de salud y falta de formación o interés de los profesionales.

## INTRODUCTION

Institutional violence (IV) against dependent older adults in Brazil requires reflections based on listening to public healthcare service managers. They are, in most cases, responsible for providing quality care to users, caregivers and families.

Population aging is a major challenge, due to the rapid growth of the social segment aged 60 and over and the lack of basic essential services aimed at them. The number of older adults is expected to exceed 1.6 billion by 2050 worldwide. In North Africa, West Asia and Sub-Saharan Africa, and in the countries of the Americas, this phenomenon will occur even more rapidly in the next three decades^(1)^.

In Brazil, the number of older adults over 60 years of age increased from 20.5 million in 2010 to 31.2 million in 2023, with an estimated 41.52 million in 2030 and 73.5 million in 2060. Therefore, there are real concerns about what public policies and society as a whole can do to improve the living conditions and health of this social segment, given that, as age increases, care needs to be specifically promoted in a country of continental dimensions like Brazil^(1-3)^.

Therefore, there is a growing demand related to social protection and healthcare, considering that, at this stage of life, there is a decrease in functional capacity due to multiple factors, the specificities of which need to be recognized from a comprehensive care perspective^(4)^. Added to this is the increased exposure to violence, especially in the segment of dependent older adults, who suffer the most from psychological violence, neglect, property abuse and physical mistreatment, both nationally^(4-7)^ and internationally^(6,8,9)^. The State, companies, civil society and families must be jointly responsible for guaranteeing the right to adequate, quality care^(4)^.

It is worth noting that, given the bottlenecks in care, the invisibility of this group’s specific needs has a direct impact on illness, physical and mental distress and social abandonment^(4,10,11)^. Age stereotypes (ageism) are reproduced in both social assistance and healthcare services, influencing the lack of sensitivity in care that often violates the constitutional rights of this more vulnerable population segment, which represents forms of IV. However, despite the absence of a consolidated national policy, there are some actions that, although still incipient, are light on the paths to be followed in favor of older adults^(4,6)^.

It reinforces the understanding that IV does not only materialize in practices of mistreatment and negligence practiced in services provided to older adults, but is also expressed in rules, hierarchical functioning, bureaucratic relationships and modes of action, and failures in assistance within institutions, in which such forms of organization and care for this population segment must be avoided^(12-14)^.

Therefore, carrying out studies on IV, particularly against dependent older adults, is timely as it enables reflection on its multiple dimensions, which are sometimes normalized in everyday life in healthcare institutions^(4,15)^. In fact, listening to managers has the potential to broaden understanding of the phenomenon from the perspective of those responsible for organizing services and ensuring the provision of care, as well as encouraging the formulation of proposals aimed at preventing and mitigating the effects of IV on dependent older adults’ lives and health.

The study delves into a specific point: what are the manifestations of IV, i.e., those produced by the system itself, against dependent older adults in primary healthcare (PHC) managers’ understanding in Brazil?

## OBJECTIVES

To analyze PHC managers’ understanding regarding the manifestations of IV against dependent older adults in Brazil.

## METHODS

### Ethical aspects

The study was approved by the *Fundação Oswaldo Cruz* Research Ethics Committee. Participants were invited to sign the Informed Consent Form, in which all informants included in data production expressed their acceptance by hand-signing. Anonymity was preserved throughout the process and through coding of interviews. To name participants, the term “manager” was used in abbreviated form (M), followed by a numeral and their geographic location (M1, Araranguá, SC; M2, Araranguá, SC, etc.).

### Study design

This study comes from the multicenter research “*Estudo situacional dos idosos dependentes que residem com suas famílias visando subsidiar uma política de atenção e de apoio aos cuidadores*”, carried out by the *Centro Latino-Americano de Estudos sobre Violência e Saúde Jorge Careli* of the National School of Public Health/*Fundação Oswaldo Cruz*, in partnership with seven Brazilian universities.

This is a qualitative study^(16)^, anchored in hermeneutics^(17)^, which followed the COnsolidated criteria for REporting Qualitative research^(18)^ recommendations. Qualitative research is highlighted by its ability to incorporate issues related to meanings and intentions in acts, relationships, structures and social transformations, in the face of socio-historical processes that permeate human subjectivity^(16)^.

Hermeneutics configures the art of understanding texts, considering human communication, always mediated by language, which expresses the way of participating in the lived world, enabling the composition of meanings^(17,19)^. In this way, the interpretations and processes arising from human relations regarding the manifestations of IV against dependent older adults in the context of social and health assistance in Brazil are based.

### Participant selection and research setting

Sixteen PHC managers and managers of specialized services in programs and policies for older adults from the participating municipalities participated.

Managers responsible for managing healthcare for older adults, both in PHC and other specific programs, which work together to ensure care for this social segment in Brazil, were included. Health managers with less than six months of experience in the position and those who were on leave due to vacation or illness were excluded.

The research was carried out in the municipalities of Araranguá (Santa Catarina), Brasília (Federal District), Fortaleza (Ceará), Manaus (Amazonas), Porto Alegre (Rio Grande do Sul), Belo Horizonte (Minas Gerais), Rio de Janeiro (Rio de Janeiro) and Teresina (Piauí). The choice of municipalities was intentional, due to the need to represent the Brazilian scenario, given the multiple territorial, economic, social, cultural and historical dimensions of each region of the country^(3)^. It is worth noting that the choice of cities was also facilitated by involving the interests of researchers and their study groups.

### Data collection and organization

The data collection period covered June to December 2019. The first stage in the study involved contacting the Municipal Health Departments of each municipality and, subsequently, scheduling an interview with health managers to facilitate the interview. The second stage consisted of applying a semi-structured interview with guiding questions on the topic in question, allowing managers to add whatever they wanted to the proposed questions.

The interviews involved questions about social, physical, mental and cognitive dependence from the perspective of healthcare and social assistance and how managers dealt with this issue. They were conducted individually, in a setting chosen by participants, conducted by professionals trained by the research team, using a guiding script and audio recording instrument (when the interviewee consented). The interviews lasted approximately 40 minutes, although the researcher offered participants more time.

After interviews were conducted, they were transcribed in full and then the database was compiled and systematized, without the use of software. The database was first constructed by and in each location studied. It was then centralized by the study’s general coordinator and opened for access by all participating researchers, allowing them to delve deeper into specific topics.

### Data analysis

Information was analyzed based on the theoretical-methodological assumptions of hermeneutics^(17)^. To this end, the researchers used participants’ statements about their role as managers as a basis for analysis, considering the institution and the provision of care to dependent older adults. In this way, the empirical material was read, classified and categorized, and the following themes emerged: (1) Situation, passivity and perpetuation of institutional violence; (2) Structural and organizational barriers that increase institutional violence; (3) Institutional violence expressed in the disarticulation of access to the care network.

## RESULTS

### Sociodemographic characterization

Sixteen managers linked to PHC and specialized services for elderly healthcare participated, ten of whom were female and six were male. Participants’ ages ranged from 34 to 58 years. Of note were the participation of five nurses, two female and one male physician, two dental surgeons, one nutritionist, one biologist, one pedagogue, one social worker, one physical education professional and one high school graduate. The period of service experience ranged from 2 to 23 years.

### Situation, passivity and perpetuation of institutional violence

The invisibility of the existence of IV in healthcare services is negatively mentioned by managers when they talk about the lack of care proposals for this specific public. The statements demonstrate a passive naturalization regarding the phenomenon of dependent aging, as if it were something common and not a topic that concerns the State in its instances of the Brazilian Health System. Here is an example that leads to reflection:

*The State is there, sovereign, just watching. So, we have to overcome this passivity! More than 20 years ago, I was called the apocalypse, because they said that I was always announcing that we were going to get old.* [It was always] *that perverse logic: are you going to invest in the old or in the child? Are you going to invest in the old or in the worker? Are you going to invest in the old or in the pregnant woman?* (M14, Belo Horizonte, MG)

Some managers recognized that care is not disseminated due to a logic of not dependent older adults’ demands and that, even with the legal apparatus, management is limited by various contexts. It was highlighted that omission or co-omission is manifested in government decrees that do not define healthcare actions and services necessary to improve the quality of life of this population group.

Others point out that the subjectivism of Brazilian legislation makes work processes unfeasible, without providing conditions for professionals to act. However, whether intentional or not, there is an invisibility of health demands. IV manifests itself in the inertia of creating means, promoting protocols and carrying out routines necessary for the priority care provided for in the Brazilian Elderly Persons Statute. Two managers expressed their views:

*I don’t see any government decree or government legislation here to improve older adults’ quality of life. The laws are very subjective* [here one could say they are abstract], *and in the end no one does anything about it.* (M5, Teresina, PI)

*She* [the older adult] *cannot move around, and cannot have easy access to the system* [she is left without the necessary care]. *We understand the needs, but we do not have the answers to meet these needs* (M10, Porto Alegre, RS)

Of course, one cannot blame only the inertia of health managers in the face of the phenomenon of aging - a bonus of contemporary humanity - nor for older adults who have lost their autonomy. It is known that longevity is the result of a combination of favorable factors that have existed since the end of the 20^th^ century and have continued to this day. The loss of autonomy brings together biological, psychological and social contingencies.

However, even in Brazil, there are examples of initiatives that can encourage replication. A proposal for a “Care Law” has just been sent to the National Congress, demonstrating that the Brazilian State is finally waking up to the needs of people who need third-party care to survive. We need to be aware of how the State, businesspeople and society, represented by parliament and civil society entities, are committed to ensuring the good lives of all Brazilians, particularly the most vulnerable.

However, the Brazilian Elderly Persons Statute itself promises priority to people over 60, noting that they often cannot wait for care. Therefore, if a policy was and still is lacking (as the proposal has just reached parliament), on the other hand, the passivity with which they view aging shows that the issue has not yet been internalized with due vehemence in the health area. Some managers say they know what is missing:

*We realize that there is a lack of preventive measures, guidance on rights and duties. Often, family caregivers* [themselves] *are already sick, which is why I said the situation is complicated. But there are other contexts that end up hindering the work, because, as I said, without the knowledge that can really help in improving, sometimes in how to treat, how and where to seek resources, not just financial ones.* (M8, Manaus, AM)

*We, as public health, cannot reach everyone. I don’t even mean everyone, but in most of these patients* [older adults], *it will happen again and again, in primary care, again and again, again and again. We don’t see any specific action for these problems.* (M2, Araranguá, SC)

### Structural and organizational barriers that increase institutional violence

Managers recognize that access to healthcare for dependent older adults is a situation that is complicated by the dependences they may have, especially for those who are physically dependent and cannot move around and go to the Basic Health Unit. The importance of home visits is evident, which are essential for older adults who have lost their autonomy. Although it must be said that primary care cannot provide the 24-hour care offered by the caregiver at home, the importance of the “Care Policy” is evident.

The limitation of the structural resources of services researched reveals an institutional organization that is still insufficient, exposing the lack of resources and the little involvement of the services in the issue.

*Access to healthcare services is complicated in the unit. If a dependent older adult is completely confined to their home, they need home visits, and then we have one day of home visits for four Family Health teams. This restricts this person’s access to the unit’s services, because we end up having visits only every 15 days.* (M11, Fortaleza, CE)

*We know that we have to invest in older adults because they are at this stage of life, but, in practice, older adults are being cared for as an adult older adult, not necessarily as an older adult.* (M14, Belo Horizonte, MG)

IV is identified in the omission of healthcare services, the absence or interruption of drug treatments, among others. Families often resort to legal proceedings to guarantee the basic rights of their older relatives:

*There is medication in basic units, but sometimes there is a shortage because this medication has to be purchased through a tender process, and older adults end up interrupting their treatment.* (M6, Brasília, FD)

*This number of older adults is increasing. There is also the whole issue of medication, as many medications are not available in primary care, so they have to go through processes to obtain this medication. Sometimes, the family cannot afford to buy it and they are left without taking the medication.* (M4, Araranguá, SC)

IV is also perceived in the structuring of healthcare services, revealing the lack of organization for return appointments for dependent older adults in PHC and other specialized services. Often, they require long waiting times and queues for procedures.

*Sometimes, care for this older adult becomes more outdated, because the interval between appointments will be longer, especially when professionals are overworked. Now, I can’t tell you exactly how long between appointments. A while ago, it was around 1 to 2 months, but every day you ask me this, I’ll answer differently.* (M16, Belo Horizonte, MG)

### Institutional violence expressed in the disarticulation of access to the care network

Care, which should be a basic right of the elderly population, especially the most dependent, when not regularly and specifically instituted and offered, is at the genesis of IV. Health managers recognize that they seek or at least try to offer health and social services as a priority for this population. However, this is not guaranteed, even though this segment has its rights protected by the Brazilian State:

*What we have done is try to offer real priority to older adults. If an older adult arrives, we try to prioritize them, but we are unable to provide universal access for all of them.* (M11, Fortaleza, CE)

*The Family Health Strategy, today, is unable to meet all this demand, and not only in primary care, but also in specialized care.* (M3, Araranguá, SC)

*As a technician, I had nothing else to do, and the department is not in a position to take on all these patients for therapy with the psychologist. Social services are not in a position to visit everyone.* (M1, Araranguá, SC)

IV is reinforced when managers understand that universal access to the essential care network is not organized for the context of chronic conditions and dependences. This disarticulation has repercussions on the elderly population’s health indicators, especially the dependent population, because, for managers, this process results in poor monitoring, which results in other health complications. This situation was clearly stated:

*The fundamental situations are not well-organized in healthcare: chronic conditions. Above all, a good portion of dependent older adults are the result of poor monitoring of hypertension and diabetes. They have sequels, most of the time, related to cerebrovascular accidents. This, in a way, also shows a health system that does not have the appropriate care model for monitoring chronic conditions.* (M12, Fortaleza, CE)

Managers bring with them feelings of frustration, whether personal or professional, showing that, no matter how much they do, the Brazilian health system still lacks:

*There is a limit to the assistance that causes us a lot of frustration. We have to pass this on to a higher authority, and then everything takes a long time, things are not immediate. We call the Public Prosecutor’s Office to try to take action against this family, but how long will that take! Will this older adult be able to cope?* (M9, Rio de Janeiro, RJ)

**Figure 1:**
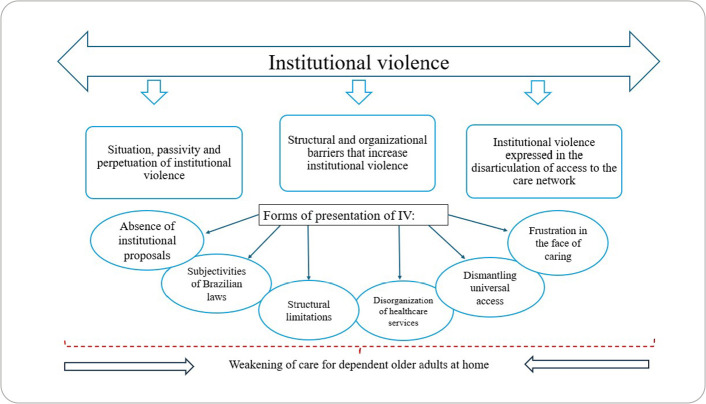
Summary of manifestations of institutional violence against dependent older adults in Brazil, 2019

Therefore, Figure 1 shows a synthetic and schematic consolidation of the manifestations of IV against dependent older adults in Brazil, based on the understandings of the managers interviewed.

## DISCUSSION

From the statements of different managers, we can see how IV is constructed in the health area and in the way of caring for older adults, especially those most dependent on care. Many of them, when asked, complain about the lack of understanding, the delays in care and the waiting lines, which greatly distress them. In a country whose imagination and concern (albeit flawed and incomplete) were children, older adults emerge as the greatest demographic novelty of the 21^st^ century, but an almost unwelcome novelty due to the ageism that contaminates society.

The perpetuation of IV emerges as a public health problem, being spread by the omission or co-omission of institutions in not offering true care to dependent older adults, in the face of constitutional barriers, the insensitivity of the State and the absence of a priority agenda for governments, due to aging and its multiple interfaces^(4,6,12,15)^.

In view of this process of relationships that permeate the human being in the context of socialization, considering the search for a social being in the historical course of life with transformations, the necessary understandings are often subordinated, making human action and experience a dependence on subordination in the face of social relations of power and mitigation of real needs^(15)^.

Thus, IV, which was never a new situation, becomes a crucial issue in this time of the century when the aging population is growing rapidly. The recent proposal for a care policy that the Ministry of Development and Social Assistance, Family and Fight against Hunger submitted to the National Congress has two sides: the person who needs assistance and the person who provides care. Both must be seen in their potential and demands, as this is the best way to prevent violence. Therefore, we also need to think about PHC services, which offer a lot and the more they offer, the more demand increases^(14,20)^.

In this context, IV is a significant barrier to compromising healthcare for older adults, even with recent advances in Brazilian public policies. This fact is consistent with the thinking of Brazilian researchers who point out that the healthcare service is considered a locus for the reproduction of IV, not restricted to a practice of forceful action, but rather to repetitive behaviors in care for older adults^(20)^.

This is the case with the increase in the number of older adults in all health facilities, in which this level of care requires permanent monitoring and assessment so that it is valued and gives its maximum potential. On the other hand, it is possible to reinvent actions, transform structures and bring proposals to life based on what we have, making primary services collaborative and powerful. There are many examples in Brazil^(5,21-23)^ and around the world^(8,9,24-26)^ that this is possible, which makes them an antidote to IV and other manifestations of violence.

Given the data and the production of knowledge, there is a need for the health manager to build facilitating strategies for coordinated action that optimizes the guarantee of care, even if this requires creativity in the face of scarce resources and everything seems invisible to society^(21,22,27)^.

It is emphasized that multidisciplinary care for this population segment in Brazil is complex, and costs are much higher than those that cover the expenses of other age groups. And the specificity of aging requires knowledge, training, humanism in care and a focus on the dependent person and the caregiver^(5)^.

Both are experiencing problems and situations that were little thought of and treated in the country just a few years ago. But problems are the material with which human beings work, transforming them into solutions. We need to move forward, preventing IV within healthcare services and building a society for all ages. Public health can and should do this.

### Study limitations

The study limitations include data production unavailability in a focus group format, the time available for managers to conduct interviews, and the limitations of the physical and organizational structures. They are also related to the selection of managers, considering only large and medium-sized municipalities in each region of the country. Despite the data heterogeneity, cultural and socioeconomic characteristics of territories, the possibility of generalization was reduced. The data transferability is also limited, i.e., the application of results to other contexts, such as small Brazilian municipalities or other countries with different health systems and standards of care practices.

### Contributions to nursing, health or public policy

The study contributes to the development of policies and strategies for the care of dependent older adults, with a view to preventing IV, or even mitigating its effects on the conditions and quality of life of this population segment. The text points out elements that enhance the reorganization of the protection network, with a view to producing comprehensive and interprofessional care.

## FINAL CONSIDERATIONS

Managers understand that IV is revealed in the complexity of the network’s operation to offer universal and comprehensive access and care to older people, especially those who depend most on the health and social sector to survive. Several factors, such as the lack of specific legislation and training on longevity, limit managers’ understanding of how to decide on care free from delays in the continuous flow of care in PHC services.

Regarding structural and organizational barriers, the lack of coordination in universal access to the health network is reflected in the difficulty that managers have in making decisions, prioritizing access, organizing home visits, and activating quality and specialized services. Therefore, the statements made by managers, which are diverse and not unanimous, show the need to seek possibilities to offer adequate and better services to older adults within what is prescribed by PHC. Such care is the best way to deal with IV.

## Data Availability

The research data are available within the article.
