# Supplementary material for: Primary Healthcare managers’ understanding of institutional violence against dependent older adults
Source: Rev Bras Enferm. 2025 Oct 3;78(Suppl 2):e20240443. doi: 10.1590/0034-7167-2024-0443 (PMC12500072; doi:10.1590/0034-7167-2024-0443)
Supplement: Translation

# Translation: Compreensão de gestores da Atenção Primária à Saúde sobre violência institucional contra pessoa idosa dependente

**DOI:** 10.1590/0034-7167-2024-0443pt

**Authors:** Jonas Loiola Gonçalves, José Maria Ximenes Guimarães, Raimunda Magalhães da Silva, Girliani Silva de Sousa, Maria Cecília de Souza Minayo, Christina César Praça Brasil, Antônio Augusto Ferreira Carioca

**Affiliations:** I Universidade Estadual do Ceará. Fortaleza, Ceará, Brasil; II Universidade de Fortaleza. Fortaleza, Ceará, Brasil; III Universidade Federal de São Paulo. São Paulo, São Paulo, Brasil; IV Fundação Oswaldo Cruz. Rio de Janeiro, Rio de Janeiro, Brasil

**Keywords:** Pessoa Idosa, Violência, Violência Contra a Pessoa Idosa, Atenção Primária à, Saúde, Gestores de Saúde.

## Abstract

**Objetivos:**

analisar a compreensão de gestores da Atenção Primária à Saúde quanto às manifestações da violência institucional contra a pessoa idosa dependente no Brasil.

**Métodos:**

estudo qualitativo, conduzido em oito cidades brasileiras. Entrevistaram-se 16 gestores de serviços de Atenção Primária à Saúde e de unidades de cuidados especializados à pessoa idosa. Para a coleta das informações, usou-se uma entrevista semiestruturada, com interpretação ancorada na análise compreensiva e crítica.

**Resultados:**

a violência institucional expressa a ausência de resposta do Estado por meio dos entraves estruturais e organizacionais potencializadores da violência, e por meio da passividade ou menosprezo dos profissionais de saúde pela pessoa idosa dependente, o que se perpetua na falta ou escassez de oferta de serviços específicos.

**Conclusões:**

a violência institucional contra a pessoa idosa dependente perpassa a passividade do Estado, barreiras estruturais, desarticulação das redes de produção de saúde e falta de formação ou interesse dos profissionais.

## INTRODUÇÃO

A violência institucional (VI) contra a pessoa idosa dependente no Brasil requer reflexões com base na escuta de gestores dos serviços públicos de saúde. Eles são, na maioria das vezes, responsáveis por fornecer a provisão de cuidados com qualidade para usuários, cuidadores e famílias.

O envelhecimento populacional é um grande desafio, devido ao crescimento acelerado do segmento social de 60 anos ou mais e à desarticulação dos serviços essenciais básicos direcionados a ele. O número de pessoas idosas deve ultrapassar 1,6 bilhão até 2050 no mundo. No norte da África, Ásia Ocidental e África Subsaariana, e nos países das Américas, esse fenômeno ocorrerá de forma ainda mais acelerada nas próximas três décadas^(1)^.

No Brasil, as pessoas idosas com mais de 60 anos passaram de 20,5 milhões, em 2010, para 31,2 milhões, em 2023, sendo estimada a marca de 41,52 milhões, em 2030, e 73,5 milhões, em 2060. São, portanto, reais as preocupações com o que as políticas públicas e a sociedade como um todo podem fazer para melhorar as condições de vida e a saúde desse segmento social, visto que, com o aumento da idade, os cuidados precisam ser especificamente promovidos em um país de dimensão continental como o Brasil^(1-3)^.

De tal modo, há uma crescente demanda relacionada à proteção social e assistência à saúde, considerando-se que, nesta fase da vida, ocorre diminuição da capacidade funcional por múltiplos fatores, cujas especificidades precisam ser reconhecidas em uma perspectiva de cuidado integral^(4)^. Acrescenta-se o aumento da exposição à violência, sobretudo no segmento das pessoas idosas dependentes, que mais sofrem violência psicológica, negligências, abusos patrimoniais e maus-tratos físicos, tanto no nacionalmente^(4-7)^ quanto internacionalmente^(6,8,9)^. Mostra-se necessária a corresponsabilidade do Estado, das empresas, da sociedade civil e das famílias na garantia do direito à assistência adequada e de qualidade^(4)^.

Destaca-se que, diante dos gargalos de cuidados, a invisibilidade das necessidades específicas desse grupo tem reflexos direto no adoecimento, no sofrimento físico e mental e no abandono social^(4,10,11)^. Encontra-se a reprodução de estereótipos da idade (etarismo) tanto nos serviços de assistência social quanto nos de saúde, influenciando a falta de sensibilidade nos atendimentos que, frequentemente, violam os direitos constitucionais desse segmento populacional mais vulnerável, o que representa formas de VI. No entanto, apesar da ausência de uma política nacional consolidada, há algumas ações que, ainda incipientes, são luz nos caminhos a serem trilhados a favor da pessoa idosa^(4,6)^.

Reforça-se o entendimento de que a VI não se concretiza somente nas práticas de maus-tratos e negligências praticadas nos serviços prestados às pessoas idosas, mas também se expressam em regras, funcionamento hierárquico, relações burocráticas e modos de ação, e falhas na assistência dentro das instituições, nas quais tais formas de organização e atendimento a esse segmento populacional devem ser evitadas^(12-14)^.

Assim, a realização de estudos sobre a VI, particularmente contra a pessoa idosa dependente, mostra-se oportuna por possibilitar a reflexão sobre suas múltiplas dimensões, por vezes normalizadas no cotidiano nas instituições de saúde^(4,15)^. Com efeito, a escuta dos gestores tem o potencial de ampliar a compreensão sobre o fenômeno sob a perspectiva daqueles que têm a responsabilidade de organizar serviços e garantir a provisão de cuidados, tal como fomentar a formulação de propostas voltadas prevenção e mitigação dos efeitos da VI na vida e na saúde das pessoas idosas dependentes.

O estudo se aprofunda em um ponto específico: quais são as manifestações de VI, ou seja, as produzidas pelo próprio sistema, contra a pessoa idosa dependente na compreensão de gestores da Atenção Primária à Saúde (APS) no Brasil?

## OBJETIVOS

Analisar a compreensão de gestores da APS quanto às manifestações da VI contra a pessoa idosa dependente no Brasil.

## MÉTODOS

### Aspectos éticos

O estudo foi aprovado pelo Comitê de Ética em Pesquisa da Fundação Oswaldo Cruz. Os participantes foram convidados a assinar o Termo de Consentimento Livre e Esclarecido, no qual todos os informantes inclusos na produção dos dados expressaram o aceite mediante assinatura manual. O anonimato foi preservado durante todo o processo e por meio de codificação das entrevistas. Para nomear os participantes, usou-se o termo “gestor” de forma abreviada (G), acrescido de um numeral e sua localização geográfica (G1, Araranguá, SC; G2, Araranguá, SC, *etc*.).

### Tipo de estudo

O presente estudo é oriundo da pesquisa multicêntrica “Estudo situacional dos idosos dependentes que residem com suas famílias visando subsidiar uma política de atenção e de apoio aos cuidadores”, realizada pelo Centro Latino-Americano de Estudos sobre Violência e Saúde Jorge Careli da Escola Nacional de Saúde Pública/Fundação Oswaldo Cruz, em parceria com sete universidades brasileiras.

Trata-se de estudo qualitativo^(16)^, ancorado na hermenêutica^(17)^, que seguiu as recomendações do *Consolidated criteria for Reporting Qualitative research* ^(18)^. A pesquisa qualitativa é destacada pela capacidade de incorporar as questões referentes aos significados e intencionalidades em atos, relações, estruturas e transformações sociais, diante dos processos sócio-históricos que permeiam a subjetividade humana^(16)^.

A hermenêutica configura a arte da compreensão de textos, considerando-se a comunicação humana, sempre mediada pela linguagem, que expressa o modo de participar do mundo vivido, possibilitando a composição de sentidos^(17,19)^. Desse modo, fundamentam-se as interpretações e processos advindos das relações humanas acerca das manifestações da VI contra a pessoa idosa dependente no contexto da assistência social e à saúde no Brasil.

### Seleção dos participantes e cenário de pesquisa

Participaram 16 gestores da APS e gestores dos serviços especializados em programas e políticas para pessoas idosas dos municípios participantes. Foram incluídos gestores responsáveis pelo gerenciamento dos cuidados de saúde da pessoa idosa tanto da APS quanto dos demais programas específicos, que se articulam na busca da garantia do cuidado a esse segmento social no Brasil. Foram excluídos os gestores de saúde com menos de seis meses de atuação no cargo e os que estavam afastados por férias ou adoecimento.

A pesquisa foi realizada nos municípios de Araranguá (Santa Catarina), Brasília (Distrito Federal), Fortaleza (Ceará), Manaus (Amazonas), Porto Alegre (Rio Grande do Sul), Belo Horizonte (Minas Gerais), Rio de Janeiro (Rio de Janeiro) e Teresina (Piauí). A escolha dos municípios foi intencional, devendo-se à necessidade de uma representação do cenário brasileiro, visto as múltiplas dimensões territoriais, econômicas, sociais, culturais e históricas de cada região do país^(3)^. Destaca-se que a escolha das cidades também foi facilitada por envolver o interesse dos pesquisadores e seus grupos de estudo.

### Coleta e organização dos dados

O período da produção dos dados abrangeu os meses de junho a dezembro de 2019. O primeiro procedimento do estudo compreendeu o contato com as Secretarias Municipais de Saúde de cada município e, posteriormente, agendamento com os gestores de saúde para viabilizar a entrevista. O segundo momento consistiu na aplicação de uma entrevista semiestruturada com perguntas norteadoras sobre a temática em foco, dando abertura aos gestores para que pudessem acrescentar o que quisessem às questões propostas.

As entrevistas envolveram questionamentos acerca da dependência social, física, mental e cognitiva do ponto de vista da atenção à saúde e da assistência social e como os gestores lidavam com essa questão. Elas foram realizadas individualmente, em ambiente escolhido pelo participante, conduzidas por profissionais capacitados e treinados pela equipe de pesquisa, usando roteiro direcionador e instrumento de gravação de áudio (quando havia consentimento do entrevistado). As entrevistas tiveram duração de aproximadamente 40 minutos, mesmo tendo o pesquisador oferecido mais tempo ao participante.

Após a realização das entrevistas, procedeu-se à sua transcrição na íntegra para, seguidamente, compor e sistematizar o banco de dados, sem apoio de *software*. O banco de dados foi construído primeiro por e em cada localidade pesquisada. Em seguida, foi centralizado pela coordenação geral do estudo e aberto para acesso de todos os pesquisadores participantes, permitindo-lhes aprofundar temas específicos.

### Análise dos dados

Analisaram-se as informações com base nos pressupostos teórico-metodológicos da hermenêutica^(17)^. Para tanto, os pesquisadores tomaram como substrato de análise as falas dos participantes sobre atuação como gestores, considerando a instituição e a provisão de cuidados às pessoas idosas dependentes. De tal modo, procedeu-se à leitura flutuante, classificação e categorização do material empírico, emergindo as seguintes temáticas: (1) Situação, passividade e perpetuação da violência institucional; (2) Entraves estruturais e organizacionais que potencializam a violência institucional; (3) A violência institucional expressa na desarticulação do acesso à rede de cuidado.

## RESULTADOS

### Caracterização sociodemográfica

Participaram 16 gestores vinculados à APS e serviços especializados para atenção à saúde da pessoa idosa, sendo dez gestores do sexo feminino e seis do sexo masculino. A idade dos participantes variou de 34 a 58 anos. Destaca-se a participação de cinco enfermeiras, duas médicas e um médico, dois cirurgiões-dentistas, uma nutricionista, uma bióloga, uma pedagoga, uma assistente social, um profissional de educação física e um de nível médio. O período de atuação no serviço variou de 2 a 23 anos.

### Situação, passividade e perpetuação da violência institucional

A invisibilidade da existência de VI nos serviços de saúde é negativamente mencionada pelos gestores, quando falam da ausência de propostas de cuidado para esse público específico. As manifestações demonstram uma naturalização passiva quanto ao fenômeno do envelhecimento dependente, como se fosse algo comum e não um tema que diz respeito ao Estado em suas instâncias do Sistema Único de Saúde. Eis um exemplo que leva à reflexão:

*O Estado está lá soberano, só olhando. Então, a gente tem de vencer essa passividade! Há mais de 20 anos, eu era chamada de apocalipse, porque eles diziam que eu só vivia anunciando que a gente ia ficar velha.* [Foi sempre] *aquela lógica perversa: você vai investir no velho ou na criança? Vai investir no velho ou no trabalhador? Vai investir no velho ou na gestante?* (G14, Belo Horizonte, MG)

Alguns gestores reconheceram que o cuidado não é difundido por uma lógica da não priorização das demandas da pessoa idosa dependente e que, mesmo com o aparato legal, a gestão é limitada por diversos contextos. Destacou-se que a omissão ou co-omissão se manifesta nos decretos governamentais que não definem ações e serviços de saúde necessários para melhorar a qualidade de vida dessa camada populacional.

Outros assinalam que os subjetivismos da legislação brasileira inviabilizam os processos de trabalho, sem dar condições para os profissionais atuarem. Mas, de propósito ou não, há uma invisibilidade das demandas de saúde. A VI se manifesta na inércia de criar meios, promover protocolos e realizar rotinas necessárias ao atendimento prioritário previsto no Estatuto da Pessoa Idosa. Dois gestores se manifestaram:

*Eu não vejo aqui* decreto de *governo, de legislação governamental para melhorar a qualidade de vida do idoso. As leis são muito subjetivas* [aqui poder-se-ia dizer que são abstratas]*, termina ninguém fazendo.* (G5, Teresina, PI)

*Ela* [pessoa idosa] *não consegue se deslocar, e não consegue ter fácil acesso ao sistema* [fica sem os cuidados necessários]*. A gente entende as necessidades, mas a gente não tem respostas para que essas necessidades sejam atendidas.* (G10, Porto Alegre, RS)

Claro que não se pode responsabilizar apenas a inércia dos gestores de saúde diante do fenômeno do envelhecimento - um bônus da humanidade contemporânea - nem pelas pessoas idosas que perderam a autonomia. Sabe-se que a longevidade é fruto de uma conjunção de fatores favoráveis que vêm do final do século XX e vêm se estendendo na atualidade. A perda de autonomia congrega contingências biológicas, psicológicas e sociais.

Porém, mesmo no Brasil, há exemplo de iniciativas que podem estimular réplicas. E acaba de ser mandada para o Congresso Nacional uma proposta de “Lei de Cuidados”, evidenciando que, por fim, o Estado brasileiro se desperta para as necessidades das pessoas que precisam de cuidados de terceiros para sobreviver. É preciso estar atento para como o Estado, os empresários e a sociedade, representada pelo parlamento e entidades da sociedade civil, curvam-se ao bom encaminhamento da vida de todos os brasileiros, particularmente os mais frágeis.

No entanto, o próprio Estatuto da Pessoa Idosa promete prioridade às pessoas acima de 60, assinalando que elas frequentemente não podem esperar pelo atendimento. Portanto, se faltava e falta ainda uma política (pois a proposta acaba de chegar ao parlamento), por outro lado, a passividade com que encaram o envelhecimento mostra que o tema ainda não foi internalizado com a devida veemência na área da saúde. Alguns gestores manifestam que sabem o que falta:

*A gente percebe que faltam medidas preventivas, orientação de direitos e deveres. Muitas vezes, os cuidadores familiares,* [eles próprios] *já estão doentes, por isso falei que é complicada a situação. Mas existem outros contextos que acabam engessando o fazer, pois, como falei, sem os conhecimentos que possa realmente ajudar na melhoria, às vezes na forma de tratar, como e onde buscar recursos, não somente financeiros.* (G8, Manaus, AM)

*A gente como saúde pública não consegue chegar em todos, não consegue chegar. Nem digo a todos, mas na maioria desses pacientes* [pessoa idosa], *vai se repetir, repetindo, na atenção básica, repetindo, repetindo, repetindo. A gente não vê uma ação específica para esses problemas.* (G2, Araranguá, SC)

### Entraves estruturais e organizacionais que potencializam a violência institucional

Os gestores reconhecem que o acesso à saúde para as pessoas idosas dependentes é uma situação que se complica em face das dependências que ela possa ter, principalmente para quem se encontra em dependência física e não consegue se locomover e comparecer à Unidade Básica de Saúde. Emana-se a importância das visitas domiciliares, fundamentais para os longevos que perderam a autonomia. Embora seja preciso dizer que a atenção básica não pode suprir os cuidados 24 horas ofertados pela pessoa cuidadora no lar, evidencia-se a importância da “Política de Cuidados”.

A limitação dos recursos estruturais dos serviços pesquisados revela uma organização institucional ainda insuficiente, expondo a falta de recursos e o pouco envolvimento dos serviços na questão.

*O acesso ao serviço de saúde é complicado na unidade. Um idoso dependente, se for totalmente restrito ao lar, ele precisa de visita domiciliar, e aí nós temos um dia de visita domiciliar para quatro equipes de Saúde da Família. Isso restringe o acesso dessa pessoa ao serviço da unidade, porque a gente acaba tendo visitas só de 15 em 15 dias.* (G11, Fortaleza, CE)

*A gente sabe que tem de investir no velho porque ele está nessa fase da vida, mas, na prática, o idoso está sendo atendido como um idoso adulto, não necessariamente como uma pessoa idosa.* (G14, Belo Horizonte, MG)

A VI se identifica na omissão dos serviços de saúde, na ausência ou na quebra de tratamentos medicamentosos, entre outros. Frequentemente, as famílias recorrem a processos de judicialização para garantir os direitos básicos de seus longevos:

*Tem medicação nas unidades básicas, mas às vezes, falta em virtude dessa medicação ter que ser comprada por intermédio de licitação, e as pessoas idosas acabam interrompendo o tratamento.* (G6, Brasília, DF)

*Cada vez aumenta mais esse número de idosos. Tem toda a parte de medicação também, que muitas medicações não têm na atenção básica, então eles têm que fazer processos para conseguir essa medicação. Às vezes, a família não tem condições de comprar e eles ficam sem tomar a medicação.* (G4, Araranguá, SC)

A VI também é percebida na estruturação dos serviços de saúde, ao revelar a falta da organização para retornos a consultas para a pessoa idosa dependente na APS e demais serviços especializados. Muitas vezes, precisam de longos tempos de espera e filas para procedimentos.

*Às vezes, o cuidado com essa pessoa idosa vai ficando mais defasado mesmo, porque o intervalo entre consultas vai ser maior, principalmente quando os profissionais estão sobrecarregados. Agora, não vou saber te precisar o tempo entre as consultas. A um tempo atrás, era em torno de 1 a 2 meses, mas cada dia que você me perguntar isso, eu vou responder de um jeito.* (G16, Belo Horizonte, MG)

### A violência institucional expressa na desarticulação do acesso à rede de cuidado

O cuidado, que deveria ser um direito básico da população idosa, principalmente a mais dependente, quando não regular e especificamente instituído e oferecido, está na gênese da VI. Os gestores de saúde reconhecem que buscam ou pelo menos tentam ofertar atendimentos de saúde e social como algo prioritário para essa população. Porém, isso não é garantido, mesmo esse segmento tenha seus direitos amparados pelo Estado brasileiro:

*O que a gente tem feito é tenta ofertar uma prioridade realmente aos idosos. Se chega um idoso, a gente já tenta priorizar, mas não conseguimos dar um acesso universal para todos eles.* (G11, Fortaleza, CE)

*A Estratégia de Saúde da Família, hoje, não consegue atender a toda essa demanda, e não é só na atenção básica, mas também na atenção especializada.* (G3, Araranguá, SC)

*Eu, enquanto técnica, não tinha mais o que fazer, e a secretaria não tem condições de ficar absorvendo todos esses pacientes para terapia com a psicóloga. A assistência social não tem condições de visitar todo mundo.* (G1, Araranguá, SC)

A VI é reforçada quando os gestores compreendem que o acesso universal à rede de cuidados essenciais não está organizado para o contexto das cronicidades e dependências. Essa desarticulação reverbera nos indicadores de saúde da população idosa, principalmente a dependente, pois, para os gestores, esse processo resulta em um mau acompanhamento, que resulta em outras complicações de saúde. Essa situação foi colocada claramente:

*As situações fundamentais não estão bem-organizadas na atenção à saúde: as condições crônicas. Principalmente uma boa parte dos idosos dependentes são resultados do mau acompanhamento de hipertensão e de diabetes. Eles têm sequelas, na maioria das vezes, relacionadas a acidente vasculares celebrais. Isso, de certa forma, mostra ainda um sistema de saúde que não está no modelo de atenção adequado para acompanhamento de condições crônicas.* (G12, Fortaleza, CE)

Os gestores trazem consigo os sentimentos de frustração, sejam pessoais ou profissionais, evidenciando que, por mais que façam, falta muito ao sistema de saúde brasileiro:

*A assistência tem um limite que nos causa muita frustração. A gente tem que passar isso para uma instância superior, e aí tudo demora, as coisas não são imediatas. Aciona-se o Ministério Público para tentar acionar essa família, mas quanto tempo isso demora! Esse idoso vai aguentar?* (G9, Rio de Janeiro, RJ)

Destarte, apresenta-se na Figura 1 uma consolidação sintética e esquemática das manifestações da VI contra a pessoa idosa dependente no Brasil, a partir das compreensões dos gestores entrevistados.

**Figura 1:** Síntese das manifestações da violência institucional contra a pessoa idosa dependente no Brasil, 2019

## DISCUSSÃO

A partir das falas dos diferentes gestores, observa-se como se constrói a VI na área da saúde e na forma de cuidar das pessoas idosas, principalmente as mais dependentes de cuidado. Muitos deles, quando perguntados, queixam-se da falta de compreensão, das demoras de atendimento e das filas de espera, que os afligem muito. Em um país cujo imaginário e cuja preocupação (ainda que falha e incompleta) eram as crianças, as pessoas idosas surgem como a maior novidade demográfica do século XXI, mas uma novidade quase indesejada devido ao etarismo que contamina a sociedade.

A perpetuação da VI desponta como um problema de saúde pública, sendo difundida pela omissão ou co-omissão das instituições em não ofertar o verdadeiro cuidado à pessoa idosa dependente, diante das barreiras constitucionais, insensibilidade do Estado e ausência de uma agenda prioritária de governos, devido ao envelhecimento e suas múltiplas interfaces^(4,6,12,15)^.

Diante desse processo de relações que permeiam o ser humano no contexto da socialização, tendo em vista a busca de um ser social no curso histórico da vida com transformações, muitas vezes subordina-se as compreensões necessárias, tornando a ação e a experiência humana uma dependência de subordinação diante das relações sociais de poder e mitigação das reais necessidades^(15)^.

Assim, a VI, que nunca foi uma situação nova, torna-se um tema crucial nesta quadra do século em que cresce aceleradamente a população que envelhece. A recente proposta de política de cuidados que o Ministério do Desenvolvimento e Assistência Social, Família e Combate à Fome entregou ao Congresso Nacional tem duas faces: a pessoa que precisa de assistência e a pessoa que cuida. Ambas devem ser vistas em suas potencialidades e demandas, sendo a melhor forma de prevenir a violência. Por isso, precisamos pensar também nos serviços de APS, que oferecem muito e quanto mais oferecem, mais a demanda aumenta^(14,20)^.

Nessa lupa, a VI é uma barreira importante para o comprometimento da assistência à saúde da pessoa idosa, mesmo com os avanços recentes das políticas públicas brasileiras. Tal fato coaduna com o pensamento de pesquisadores brasileiros que apontam que o serviço de saúde é considerado um lócus para a reprodução da VI, não restringindo a uma prática de ação da força, mas sim a condutas de repetição na assistência à pessoa idosa^(20)^.

É o caso da elevação do número de pessoas idosas em todos os equipamentos de saúde, no qual esse nível de atenção necessita de monitoramento permanente e avaliação, para que seja valorizado e dê o máximo de suas potencialidades. Por outro lado, é possível reinventar ações, transformar estruturas e tornar vivas as propostas a partir do que se tem, tornando os serviços primários colaborativos e potentes. Há muitos exemplos no Brasil^(5,21-23)^ e no mundo^(8,9,24-26)^ de que é possível, o que se os torna um antídoto contra a VI e outras manifestações da violência.

Diante dos dados e da produção do conhecimento, há necessidade de o gestor de saúde construir estratégias facilitadoras para uma atuação articulada que otimize a garantia do cuidado, ainda que isso exija criatividade diante dos parcos meios e tudo pareça invisível à sociedade^(21,22,27)^.

Enfatiza-se que o cuidado multiprofissional para esse segmento populacional no Brasil é complexo, e os custos são muito mais elevados do que os que recobrem os gastos de outras faixas etárias. E a especificidade do envelhecimento exige conhecimento, formação, humanismo no atendimento e foco na pessoa dependente e no cuidador^(5)^.

Ambos vivenciam problemas e situações pouco pensadas e tratadas no país poucos anos atrás. Mas os problemas são o material com o qual os seres humanos trabalham, transformando-os em solução. É preciso ir em frente, prevenindo a VI dentro dos serviços de saúde e construindo uma sociedade para todas as idades. A saúde coletiva pode e deve fazer isso.

### Limitações do estudo

As limitações do estudo perpassam a indisponibilidade da produção dos dados em caráter de grupo focal, tempo disposto pelos gestores para a realização das entrevistas e as limitações das estruturas físicas e organizacionais. Estão relacionadas também à seleção dos gestores, considerando-se somente municípios de grande e médio porte em cada região do país. Apesar da heterogeneidade dos dados, características culturais e socioeconômicas dos territórios, reduziu-se a possibilidade de generalização. A transferibilidade dos dados também é limitada, ou seja, a aplicação dos resultados a outros contextos, como pequenos municípios brasileiros ou outros países com sistemas de saúde e padrões de práticas assistenciais distintos.

### Contribuições para as áreas da enfermagem, saúde ou políticas públicas

O estudo contribui para a elaboração do desenho de políticas e estratégias de cuidados com as pessoas idosas dependentes, com vistas à prevenção da VI, ou ainda, à mitigação de seus efeitos nas condições e qualidade de vida desse seguimento populacional. O texto aponta elementos que potencializam a reorganização da rede de proteção, com vistas à produção do cuidado integral e interprofissional.

## CONSIDERAÇÕES FINAIS

Os gestores compreendem que a VI é revelada na complexidade da operação da rede para ofertar acesso e cuidado universal e integral às pessoas idosas, principalmente àquelas que mais dependem do setor de saúde e social para sobreviver. Vários fatores como falta de legislação específica e de formação sobre a longevidade limitam a compreensão dos gestores para decidir sobre um cuidado livre de retardos no fluxo contínuo de atendimento nos serviços da APS.

Em relação aos entraves estruturais e organizacionais, a desarticulação do acesso universal à rede de saúde se reflete na dificuldade do gestor tomar decisões, priorizar acesso, organizar visitas ao domicílio e acionar serviços de qualidade e especializados. Portanto, as manifestações dos gestores, que são diversas e não unânimes, mostram a necessidade de buscar possibilidades para ofertar os adequados e melhores serviços às pessoas idosas dentro do que é prescrito pela APS. Esses cuidados são a melhor forma de enfrentamento da VI.

## Data Availability

Os dados da pesquisa estão disponíveis no corpo do artigo.
